# Aged xCT-Deficient Mice Are Less Susceptible for Lactacystin-, but Not 1-Methyl-4-Phenyl-1,2,3,6- Tetrahydropyridine-, Induced Degeneration of the Nigrostriatal Pathway

**DOI:** 10.3389/fncel.2021.796635

**Published:** 2021-12-17

**Authors:** Eduard Bentea, Laura De Pauw, Lise Verbruggen, Lila C. Winfrey, Lauren Deneyer, Cynthia Moore, Giulia Albertini, Hideyo Sato, Ann Van Eeckhaut, Charles K. Meshul, Ann Massie

**Affiliations:** ^1^Laboratory of Neuro-Aging and Viro-Immunotherapy, Vrije Universiteit Brussel, Brussels, Belgium; ^2^Neurocytology Laboratory, Veterans Affairs Medical Center, Research Services, Portland, OR, United States; ^3^Department of Medical Technology, Niigata University, Niigata, Japan; ^4^Research Group Experimental Pharmacology, Department of Pharmaceutical Chemistry, Drug Analysis and Drug Information, Vrije Universiteit Brussel, Brussels, Belgium; ^5^Department of Behavioral Neuroscience and Pathology, Oregon Health and Science University, Portland, OR, United States

**Keywords:** glutamate, neuroprotection, aging, proteasome inhibition, Parkinson’s disease

## Abstract

The astrocytic cystine/glutamate antiporter system x_*c*_^–^ (with xCT as the specific subunit) imports cystine in exchange for glutamate and has been shown to interact with multiple pathways in the brain that are dysregulated in age-related neurological disorders, including glutamate homeostasis, redox balance, and neuroinflammation. In the current study, we investigated the effect of genetic xCT deletion on lactacystin (LAC)- and 1-methyl-4-phenyl-1,2,3,6-tetrahydropyridine (MPTP)-induced degeneration of the nigrostriatal pathway, as models for Parkinson’s disease (PD). Dopaminergic neurons of adult xCT knock-out mice (xCT^–/–^) demonstrated an equal susceptibility to intranigral injection of the proteasome inhibitor LAC, as their wild-type (xCT^+/+^) littermates. Contrary to adult mice, aged xCT^–/–^ mice showed a significant decrease in LAC-induced degeneration of nigral dopaminergic neurons, depletion of striatal dopamine (DA) and neuroinflammatory reaction, compared to age-matched xCT^+/+^ littermates. Given this age-related protection, we further investigated the sensitivity of aged xCT^–/–^ mice to chronic and progressive MPTP treatment. However, in accordance with our previous observations in adult mice (Bentea et al., 2015a), xCT deletion did not confer protection against MPTP-induced nigrostriatal degeneration in aged mice. We observed an increased loss of nigral dopaminergic neurons, but equal striatal DA denervation, in MPTP-treated aged xCT^–/–^ mice when compared to age-matched xCT^+/+^ littermates. To conclude, we reveal age-related protection against proteasome inhibition-induced nigrostriatal degeneration in xCT^–/–^ mice, while xCT deletion failed to protect nigral dopaminergic neurons of aged mice against MPTP-induced toxicity. Our findings thereby provide new insights into the role of system x_*c*_^–^ in mechanisms of dopaminergic cell loss and its interaction with aging.

## Introduction

The cystine/glutamate antiporter system x_*c*_^–^ is an astrocytic plasma membrane antiporter (Ottestad-Hansen et al., 2018) that couples the export of glutamate to the import of cystine in an equimolar ratio (Sato et al., 2005). System x_*c*_^–^ functions as a heterodimer, in which the specific subunit xCT (encoded by the gene *Slc7a11*) mediates the transport function of the antiporter, while 4F2hc functions as a molecular chaperone anchoring xCT to the plasma membrane (Massie et al., 2015).

After reaching the cytosol, cystine imported by system x_*c*_^–^ is rapidly reduced to cysteine, an essential substrate in the synthesis of glutathione. Furthermore, cystine delivered *via* system x_*c*_^–^ can modulate the extracellular cysteine/cystine redox couple after being shuttled *via* system Alanine-Serine-Cysteine (ASC) or system L (Lewerenz et al., 2013). Importantly, while *in vitro* cells are dependent on system x_*c*_^–^ for intracellular glutathione homeostasis and survival (Sato et al., 2005), no change in glutathione levels or signs of oxidative stress have been reported in the brain of xCT-deficient mice (De Bundel et al., 2011; Massie et al., 2011; Dang et al., 2017), indicating that cells may be less dependent on system x_*c*_^–^ for basal glutathione production *in vivo*.

In addition to its role in regulating redox function, system x_*c*_^–^ provides up to 70% of the extracellular glutamate levels in areas of the brain such as the hippocampus (De Bundel et al., 2011) and striatum (Massie et al., 2011). Accumulating evidence supports the extrasynaptic location of system x_*c*_^–^ and glutamate released by system x_*c*_^–^ will thus act on extrasynaptic ionotropic and metabotropic glutamate receptors to fine-tune excitatory neurotransmission at the neuron-glia interface (Baker et al., 2002; Bridges et al., 2012; Soria et al., 2014). Consistent with this, pharmacological or genetic modulation of the antiporter has been shown to modulate various synaptic circuits in the brain, including cortico-striatal (Bentea et al., 2021), cortico-accumbens (Moran et al., 2005), and hippocampal CA3-CA1 synapses (Williams and Featherstone, 2014). At the same time, excessive extrasynaptic glutamate levels can be toxic *via* overstimulation of ionotropic glutamate receptors (Hardingham and Bading, 2010), a pathway of neuronal stress and toxicity -i.e., excitotoxicity- involved in various neurodegenerative disorders including Parkinson’s disease (PD) (Ambrosi et al., 2014).

Previous findings suggest an involvement of system x_*c*_^–^ in pathogenic pathways leading to degeneration of nigral dopaminergic neurons. Expression of xCT is affected in PD models, although findings have been inconsistent, depending on the brain region investigated, model employed, and time point post-lesion evaluated (Massie et al., 2008; Bentea et al., 2015a,2017a; El Arfani et al., 2015). Moreover, genetic deletion of xCT was neuroprotective in the 6-hydroxydopamine (6-OHDA) mouse model of PD, as evidenced by decreased nigral cell loss following intra-striatal administration of the dopaminergic toxin in adult and aged xCT knock-out (xCT^–/–^) mice vs. age-matched wild-type (xCT^+/+^) littermates (Massie et al., 2011). Similarly, genetic deletion of xCT or pharmacological inhibition of system x_*c*_^–^ was found to be protective against metamphetamine-induced dopaminergic neurotoxicity and microglial activation (Dang et al., 2017). On the other hand, xCT deletion did not affect nigrostriatal degeneration induced by systemic administration of MPTP in adult mice, suggesting that the neuroprotective effects may be dependent on the mechanisms of toxicity (Bentea et al., 2015a).

To further obtain insight into the neuroprotective potential of targeting system x_*c*_^–^ in PD, we here investigated the link between system x_*c*_^–^ and proteasome inhibition-induced neurodegeneration. Recent studies revealed structural and functional deficits in the ubiquitin-proteasome pathway in PD, suggesting that impaired proteostasis plays an important role in the pathogenesis (Lehtonen et al., 2019). In line with this, administration of proteasome inhibitors, such as lactacystin (LAC), to the nigrostriatal pathway in rodents replicates features characteristic of PD, including nigrostriatal degeneration and deficits in motor function (Bentea et al., 2017b). While this mechanism has been acknowledged to play an important role in the pathogenesis, targets affecting this pathway may be unique in their profile when compared to other classical PD toxin-based models (Konieczny et al., 2014; Harrison et al., 2019), allowing for a better understanding of the mechanisms of cell loss in PD. Previously, expression of xCT was found to be increased following proteasome inhibition in T24 bladder carcinoma cells, indicating that its levels may be controlled *via* proteasomal degradation of its upstream transcription factors Nrf2 and ATF4 (Ye et al., 2014). In addition, a toxic synergistic interplay has been reported between glutamate excitotoxicity and proteasome inhibition, with extrasynaptic NMDA receptors preferentially coupled to the downregulation of the proteasome system (Caldeira et al., 2013). All together, these findings suggest that modulation of extrasynaptic glutamate may influence proteasome inhibition-induced toxicity and prompted us to study the susceptibility of xCT-deficient mice to LAC-induced neurodegeneration. As aging represents the main risk factor for PD (Abdullah et al., 2015) and to improve translatability of our findings, both adult and aged mice were studied.

Next, we investigating whether aged xCT-deficient mice are protected in the chronic and progressive MPTP model. As for the interplay with proteasome inhibition, an interaction between glutamate excitotoxicity/extrasynaptic NMDA activation and mitochondrial dysfunction has been identified (Gouix et al., 2009; Stanika et al., 2009), whereas mitochondrial dysfunction elicited by the active metabolite of MPTP, MPP+, upregulates the expression of xCT in LUHMES neurons (Tong et al., 2020). Although we did not reveal a differential effect of xCT deletion in the MPTP model in adult mice (Bentea et al., 2015a), aging sensitizes neurons to the toxicity of glutamate (Brewer, 1998; Brewer et al., 2005), possibly due to age-related depolarization of the mitochondrial membrane potential and increased production of mitochondrial reactive oxygen species (Parihar and Brewer, 2007), prompting us to investigate a possible link between xCT and MPTP-induced neurotoxicity in aged animals.

In the current study we reveal age-dependent protection of xCT^–/–^ mice against LAC-induced nigrostriatal degeneration. However, in accordance to our previous observations in adult mice, xCT deletion did not confer any protection against MPTP-induced toxicity.

## Materials and Methods

### xCT-Deficient Mouse Model

xCT^–/–^ and xCT^+/+^ littermates were obtained from a colony of heterozygous high-generation descendants of the strain described previously by Sato et al. (2005). The xCT null mutants were generated by targeted disruption of the START codon in exon 1 of the *Slc7a11* gene and were backcrossed for more than 12 generations on a C57BL/6J background. Genotyping of xCT mutant mice was performed by PCR amplification of ear punch DNA using the REDExtract-N-Amp Tissue PCR Kit (Sigma-Aldrich), and the following primers: 5′-GATGCCCTTCAGCTCGATGCGGTTCACCAG-3′ (GFPR3); 5′-CAGAGCAGCCCTAAGGCACTTTCC-3′ (mxCT5′flankF6); 5′-CCGATGACGCTGCCGATGATGATGG-3′ [mxCT(Dr4)R8].

For investigating the susceptibility of xCT-deficient mice in the LAC model, adult (3–4 months old) and aged (19–23 months old) male xCT^–/–^ and xCT^+/+^ littermates were used. Mice were bred and group-housed under standardized conditions (25°C, 10/14 h dark/light cycle), with free access to food and water, in the animal facilities of the Vrije Universiteit Brussel. Studies were performed according to national guidelines on animal experimentation and were approved by the Ethical Committee for Animal Experimentation of the Vrije Universiteit Brussel.

For investigating the susceptibility of xCT-deficient mice in the MPTP model, aged (17 months old) male xCT^–/–^ and xCT^+/+^ littermates were used. Mice originating from the colony of the Vrije Universiteit Brussel were bred at Janvier Laboratories (France) and shipped to the animal facility of the Veterans Affairs Medical Center (Portland, Oregon) to age. Mice were housed under standardized conditions, on a 12/12 h light/dark cycle, with food and water available *ad libitum*. All procedures were carried out in accordance with the National Institutes of Health (NIH) Guide for the Care and Use of Laboratory Animals and were approved by the Portland VA Medical Center Institutional Animal Care and Use Committee.

### Stereotaxic Administration of Lactacystin

Mice were anesthetized with a mixture of ketamine (100 mg/kg i.p.; Ketamine 1000 Ceva, Ceva Sante Animale) and xylazine (10 mg/kg i.p.; Rompun 2%, Bayer N.V.), and positioned in a Kopf Model 963 Ultra Precise Small Animal Stereotaxic Frame, with a mouse adaptor (David Kopf Instruments). The skull was exposed, and a small hole was made through the skull above the left substantia nigra (SN) pars compacta (SNc). A volume of 1.5 μL of 2 μg/μL LAC was injected into the left SNc at the following coordinates: anterior-posterior (AP) −3.0, medial-lateral (ML) −1.0, dorsal-ventral (DV) −4.5 from bregma (Paxinos and Franklin, 2004). To minimize lesion variability due to *ex vivo* degradation of the toxin which can yield an inactive LAC analog (Dick et al., 1996), fresh LAC solutions were prepared for every four mice by dissolving 50 μg LAC (Cayman Chemicals) in 25 μL NaCl 0.9%, and immediately stored on ice. The same batch of LAC was used to lesion all animals within each age group. Control sham-operated mice received the same volume of vehicle (NaCl 0.9%), at the same coordinates. To minimize unspecific tissue damage, microinjections were performed using a 10 μL Model 1701 RN Neuros Syringe (Hamilton Company), at a flow rate of 0.5 μL/min. After injection, the syringe was left in place for an additional 5 min, and then slowly removed. At the end of the surgery, the skin was sutured, and mice received 4 mg/kg ketoprofen i.p. (Ketofen, Merial) for post-operative analgesia.

Motor function of adult mice was investigated (rotarod test) at 1–3 weeks post-stereotaxic surgery. All mice were sacrificed at 3 weeks post-surgery by cervical dislocation. The caudal part of the brains was fixed in 4% paraformaldehyde for immunohistochemistry (IHC), while striata were dissected from the rostral part of the brain for analysis of dopamine (DA) and 3,4-dihydroxyphenylacetic acid (DOPAC) content.

### 1-Methyl-4-Phenyl-1,2,3,6-Tetrahydropyridine Treatment Paradigm

Aged mice were administered progressively increasing doses of MPTP (dissolved in saline; 0.9% NaCl) at a frequency of one i.p. injection daily for 5 days/week, according to a previously designed protocol (Hood et al., 2016). The first week, mice received a dose of 10 mg/kg, followed by 20 mg/kg and eventually 24 mg/kg during the final week of injections. Due to the high mortality of the mice during the third week of injections, partly induced by their frailty because of their age, the MPTP treatment paradigm was stopped after the third week, and the mice received five injections less compared to our previous protocol (Hood et al., 2016). Vehicle groups received daily saline injections (1 mL/kg).

One week after the final MPTP injection, mice were tested behaviorally using the DigiGait apparatus to analyze gait differences. Next, half of the animals of each group were transcardially perfused for IHC and the brains of the other half of the mice were dissected and snap frozen for DA analysis and immunoblotting.

### Behavioral Assessment

For detecting LAC-induced motor deficits, we used an accelerating rotarod system (TSE RotaRod Advanced, TSE Systems), as described previously (Bentea et al., 2015b). Prior to surgery, mice were trained for 5 min at a constant speed of 5 rpm. During this initial training phase, mice were placed immediately back on the rod after falling, allowing them to get familiarized to the test. In the second phase of training, mice underwent three repeated trials of 1 min at a fixed speed of 5 rpm, with 3 min of rest between trials. For testing the rotarod performance at baseline and after lesion, mice underwent five repeated trials that started at constant speed of 5 rpm for 30 s and continued with a 5–25 rpm accelerating protocol during 200 s, leading to a maximum total rod time of 230 s. Mice were allowed 3 min of rest between trials. The mean of the five test trials underwent statistical analysis.

MPTP-induced motor impairment was assessed using the DigiGait apparatus (Mouse Specifics, Inc.), starting 7 days following the last injection, as previously described (Goldberg et al., 2011; Hood et al., 2016; Churchill et al., 2019; Massaquoi et al., 2020). The gait of each mouse was captured by ventral plane videography through a transparent, motor-driven treadmill belt. Digital images of the paws of each mouse were taken at 150 frames/s while the mice ran at a velocity of 24 cm/s. The area of the underside of each paw relative to the area of the treadmill belt at each frame was used for spatial and temporal measurements. Data were analyzed using DigiGait Analysis 15 software. Animals that ran less than 5 s on the treadmill were excluded from the analysis.

### Immunohistochemistry

#### Immunodetection and Quantification of Nigral Dopaminergic Neurons and Microglial Cells Following Lactacystin

Forty micrometer vibratome sections were cut from the post-fixed caudal part of the brain (Leica Microsystems) and stored in serial order in 10 mM phosphate buffer saline (PBS) supplemented with 1.5 mM sodium azide at 4°C. Sections of the SN were selected to quantify the presence of dopaminergic neurons and microglial cells, using rabbit anti-TH antibody (AB152, Millipore; 1/2,000 in Tris-buffered saline, incubation overnight at room temperature) and rabbit anti-mouse Iba-1 antibody (019-19741, Wako Pure Chemicals; 1/1,000 in 20% pre-immune goat serum, incubation overnight at 4°C), respectively, and employing the ABC peroxidase technique as described previously (Bentea et al., 2015b). Immunoreactivity was visualized using 3,3’-diaminobenzidine as chromogen. Photomicrographs were taken of the stained sections, and cell counts were performed using ImageJ software (NIH). The total number of TH + profiles in the ipsi- and contralateral SNc was counted by an investigator blinded to treatment in six serial sections throughout the entire rostro-caudal extent of this brain region (AP – 2.92 to – 3.64 from bregma). The cell number and morphology of Iba-1 + cells were evaluated blindly in three representative 150 × 150 μm squares covering the entire width of the SN of three serial sections spanning the whole SN. Morphological analysis of Iba-1 + cells was performed as described previously (Albertini et al., 2018) by measuring the cell area and Feret’s diameter (the longest distance between any two points of the selected region of interest) using ImageJ.

#### Immunodetection and Quantification of Nigral Dopaminergic Neurons and Striatal Dopaminergic Terminals Following 1-Methyl-4-Phenyl-1,2,3,6-Tetrahydropyridine

Immunohistochemistry following MPTP injection was performed as described previously (Bentea et al., 2015a). MPTP-treated mice were euthanized using a solution of 1% ketamine/0.1% xylazine (20 mL/kg, i.p.) after which they were transcardially perfused with 2.5% glutaraldehyde/0.5% paraformaldehyde/0.1% picric acid. Brains were removed and cut in half coronally at the level of the hypothalamus. Both halves were placed in the same fixative and further fixed in a microwave tissue processor (Pelco BioWave, Ted Pella, Inc.), containing a temperature controlled fixation bath using a thermoelectric recirculating chiller (Pelco Steady Temp Pro, Ted Pella, Inc.) for a total of 30 min [20 min, 150 watts (W) at 28°C/10 min, 650 W at 25°C], as previously described (Xu et al., 2019; Moore et al., 2021). Brain halves were then rinsed and left in 0.1 M PBS at 4°C until being serially sectioned through the striatum (starting at bregma + 1.2 mm and ending at the level of the anterior commissure, + 0.25 mm) at 60 μm thickness, and through the entire rostral-caudal extent of the SN (AP from bregma, −2.50 to −4.24 mm) at 40 μm thickness, using a vibratome (Leica vibratome, Leica Microsystems). Every third slice of the SN or striatum was collected, resulting in a total of six slices per region per mouse. Slices were immuno-labeled using the Pelco BioWave^®^ Pro (Ted Pella Inc.) as described previously (Goldberg et al., 2011; Xu et al., 2019; Moore et al., 2021), using a primary mouse monoclonal antibody for TH (1/250 dilution, Immunostar), a secondary biotinylated goat anti-mouse antibody (1/400 dilution, Jackson ImmunoResearch), and the ABC peroxidase technique. Mounted slices at the level of the SN were counterstained with Cresyl Violet (0.2% in H_2_O) and imaged at a magnification of 5× using a Zeiss Axioplan (Carl Zeiss) and a Microbrightfield (MBF) camera and software setup (MBF Bioscience). TH + cells were counted using the ImagePro Software (ImagePro 6.3, Media Cybernetics, Inc.). Cell numbers for each side were added together and the average number of TH + cells/slice was calculated across the six slices, as previously described (Churchill et al., 2019; Massaquoi et al., 2020). Slices containing the striatum were imaged for optical density measurement at 1.25× magnification (numerical aperture of 0.035) and analyzed using ImagePro software. Both the left and right side of the brain was analyzed by subtracting the background (optical density of overlying cortex) for each side, averaging the optical density per slice, then taking the mean from all slices per mouse and eventually by making an overall mean for each group of mice.

### Western Blotting

Striatal and nigral tissue of MPTP-treated mice was homogenized in 300 μL extraction buffer [2% sodium dodecyl sulfate (SDS), 60 mM Tris, 100 mM DTT, with phosphatase and protease inhibitor cocktails (Sigma–Aldrich), pH 7.5]. Samples were incubated for 30 min at 37°C and centrifuged for 10 min at 9,500 g at 4°C. Supernatants were stored at –20°C. Protein concentrations were assessed using a fluorometric method (Qubit, Invitrogen). Equal concentrations of protein were loaded on a 4–12% gel (Criterion XT Bis-Tris Precast Gels, Bio-Rad Laboratories) and separated by SDS-polyacrylamide gel electrophoresis (PAGE) under reducing conditions (200 V, 200 mA, 25 W, 45 min). Next, proteins were transferred to a polyvinylidene fluoride membrane using the Trans-Blot Turbo Transfer System (Bio-Rad Laboratories). Non-specific binding was blocked by incubating the membranes for 1 h at room temperature in 5% enhanced chemiluminescence (ECL) Advance Membrane Blocking Agent (Cytiva Life Sciences, Amersham) before overnight incubation with rabbit polyclonal anti-TH (AB152, 1/2,000, diluted in blocking agent at room temperature), or anti-Iba-1 (019-19741, 1/1,000, diluted in blocking agent at 4°C). The following day, membranes were incubated at room temperature for 30 min with horse-radish-peroxidase conjugated anti-rabbit Immunoglobulin G antiserum (1/4,000 for TH; 1/25,000 for Iba-1; DakoCytomation). Immunoreactive proteins were visualized using ECL Prime (Cytiva Life Sciences). After immunodetection, membranes were washed overnight. The next day, membranes were incubated in stripping buffer (0.78% beta-mercaptoethanol, 2% SDS, 62.5 mM Tris, pH 6.7), after which a ServaPurple total protein stain was performed following the manufacturer’s instructions (SERVA Electrophoresis GmbH). Densitometric analysis of the immunoreactive bands was performed using the ImageQuant LAS4000 software (Cytiva Life Sciences Amersham). Densities of immunoreactive bands were normalized to the densities of the total protein stain detected on the same membrane. All immunoblots were repeated at least once, resulting in identical results.

### Neurochemical Analysis

For the analysis of DA and DOPAC content, dissected striata were weighed and homogenized in 400 μL antioxidant solution (0.05 M HCl, 0.5% Na_2_S_2_O_5_, and 0.05% Na_2_EDTA) containing 100 ng/mL 3,4-dihydroxybenzylamine as internal standard. Homogenates were centrifuged for 20 min at 10,000 × g at 4°C. Supernatants were diluted 1:5 in 0.5 M acetic acid and 20 μL of this sample dilution was analyzed for DA and DOPAC content on a narrow-bore (XBridge C18, 3.5 μm, 2.1 × 150 mm; Waters) liquid chromatography system with an electrochemical detector (Antec), as described previously (Massie et al., 2011).

### Statistical Analysis

Data are expressed as mean ± s.e.m. Statistical analyses were performed using GraphPad Prism 9.0.1 software, using two-way ANOVA followed by Tukey *post hoc* tests. The α-value was set at 0.05.

## Results

### xCT Deletion Has No Effect on Lactacystin-Induced Nigrostriatal Degeneration in Adult Mice

To evaluate whether genetic deletion of xCT influences proteasome inhibition-induced nigrostriatal degeneration in adult mice, 3–4 months old xCT^–/–^ and xCT^+/+^ littermates were stereotaxically injected with LAC in the left SNc. After behavioral analysis, various markers of the nigrostriatal pathway were comparatively assessed at the ipsi- and contralateral sides.

**FIGURE 1:**
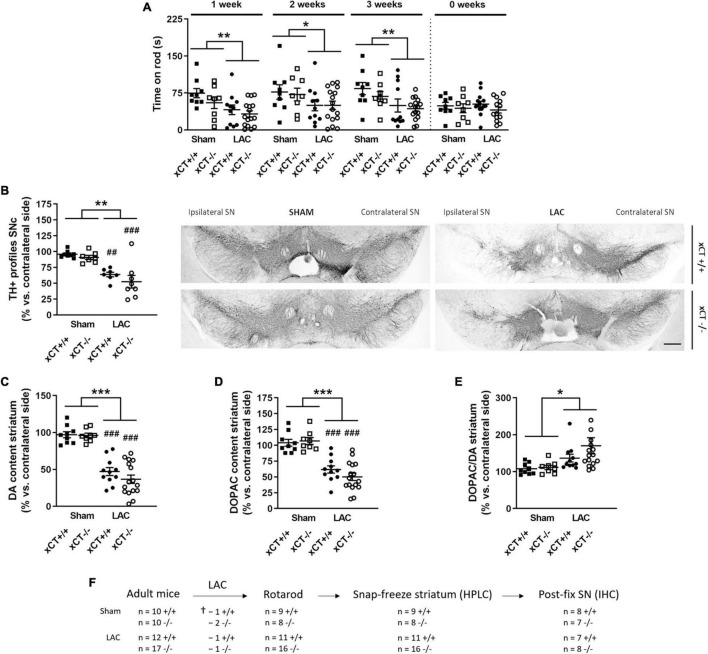
xCT deletion does not influence nigrostriatal dopaminergic degeneration or motor impairment, at 3 weeks after LAC administration in adult mice. **(A)** The rotarod test indicated a decrease in motor coordination and balance induced by LAC lesion in both xCT^– /–^ and xCT^+/+^ mice. **(B)** TH immunohistochemistry revealed a global reduction in the ipsilateral vs. contralateral ratio in the number of nigral dopaminergic neurons following LAC, to a similar extent in xCT^– /–^ and xCT^+/+^ mice. **(C)** Likewise, the loss of DA content in the ipsilateral vs. contralateral striatum following LAC was comparable between xCT^– /–^ and xCT^+/+^ mice. **(D,E)** LAC injection led to an equivalent loss of striatal DOPAC content between the genotypes **(D)** and an increase in the striatal DOPAC/DA ratio **(E)**. Data are presented as mean + s.e.m. *p < 0.05, **p < 0.01, ***p < 0.001 (two-way ANOVA, lesion effect), ^##^*p* < 0.01, ^###^*p* < 0.001 (Tukey *post hoc* vs. corresponding sham group). **(F)** Experimental design and sample size allocation (also applies to Figure 3); ^†^indicates mortality post-surgery; *n* = 8–16 mice/group in **(A)**, *n* = 7–8 mice/group in **(B)**, *n* = 8–16 mice/group in **(C–E)**. DA, dopamine; DOPAC, 3,4-dihydroxyphenylacetic acid; HPLC, high-performance liquid chromatography; IHC, immunohistochemistry; LAC, lactacystin; SN, substantia nigra; SNc, substantia nigra pars compacta; TH, tyrosine hydroxylase. Scale bar 400 μm.

Behavioral evaluation of the adult mice revealed a LAC-induced acute loss of motor function as assessed using the rotarod test, that could be observed already at 1 week post-surgery in both genotypes [lesion factor: *F*_(1,__40)_ = 9.74, *p* = 0.003], and did not progress until 3 weeks post-surgery [lesion factor: *F*_(1,__40)_ = 8.38, *p* = 0.006] (Figure 1A). This acute and non-progressive time-course of LAC-induced behavioral deficit is in line with previous reported data (Bentea et al., 2015b), and indicates an acute effect of the toxin on nigral dopaminergic neurons. As we initially planned to perform rotarod analysis on aged mice as well, we decided to continue our experiments 3 weeks post-lesion to ensure these mice would be sufficiently recovered after surgery.

Intranigral administration of LAC led to an overall reduction in the ratio of TH + profiles in the ipsilateral vs. contralateral SNc of both genotypes [lesion factor: *F*_(1,__26)_ = 36.07, *p* < 0.0001], with a significant decrease in both xCT^+/+^ (*p* = 0.003) and xCT^–/–^ mice (*p* = 0.0005), when compared with their corresponding sham-injected mice (Figure 1B). Similarly, LAC administration induced an overall reduction of DA content in the ipsilateral vs. contralateral striatum of lesioned mice, that could be observed to a similar extent in both genotypes [lesion factor: *F*_(1,__40)_ = 98.76, *p* < 0.0001] (Figure 1C). The loss of DA was mirrored by a general decrease in the content of DOPAC in the lesioned mice of both genotypes [lesion factor: *F*_(1,__40)_ = 65.74, *p* < 0.0001] (Figure 1D). The resulting increased DOPAC/DA ratio [lesion factor: *F*_(1,__40)_ = 6.09, *p* = 0.018] (Figure 1E) indicates a compensatory reaction following the lesion.

### Aged Mice Lacking xCT Show Reduced Susceptibility to Lactacystin-Induced Nigral Dopaminergic Neurodegeneration

To evaluate the impact of aging in the LAC model, aged (19–23 months old) xCT^–/–^ and xCT^+/+^ mice were intranigrally injected with LAC and characterized in terms of nigrostriatal degeneration at 3 weeks post-surgery.

Similar to the adult mice, we have attempted to evaluate motor function of the aged mice using the rotarod test. However, using the current protocol, we were unable to obtain a consistent baseline performance prior to the surgery and were not able to pursue this test as a lesion-induced outcome.

**FIGURE 2:**
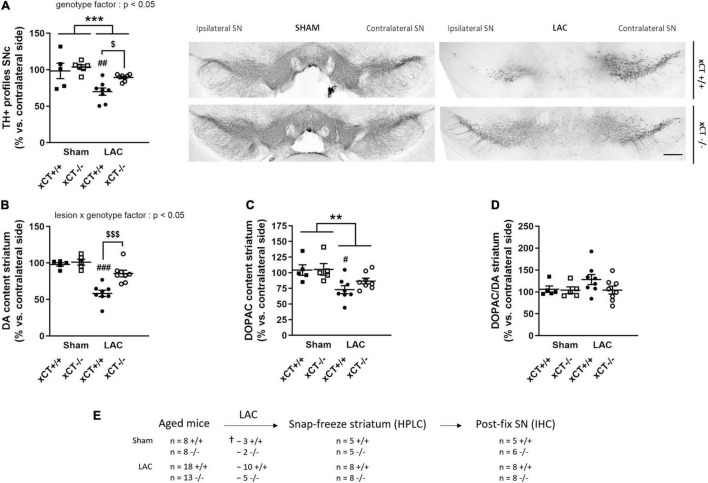
xCT deletion protects the nigrostriatal dopaminergic pathway of aged mice against LAC-induced degeneration. **(A)** TH immunohistochemistry revealed a global loss of nigral TH + profiles following LAC that was driven by a significant decrease of dopaminergic neurons in xCT^+/+^, but not xCT^– /–^ mice, as well as a significant increase in the number of TH + profiles in LAC-lesioned xCT^– /–^ vs. xCT^+/+^ mice. **(B)** LAC significantly decreased DA content in xCT^+/+^, but not xCT^– /–^, mice, with a significant difference in the % DA loss between the two genotypes. **(C)** Similarly, LAC injection led to a loss of striatal DOPAC levels in xCT^+/+^, an effect that was absent in xCT^– /–^ mice. **(D)** No change could be observed in the striatal DOPAC/DA ratio following lesion. Data are presented as mean + s.e.m. **p < 0.01, ***p < 0.001 (two-way ANOVA, lesion effect), ^#^*p* < 0.05,^ ##^*p* < 0.01, ^###^*p* < 0.001 (Tukey *post hoc* vs. corresponding sham group), ^$^*p* < 0.05, ^$$$^*p* < 0.001 (Tukey *post hoc* comparing LAC xCT^+/+^ vs. LAC xCT^– /–^). **(E)** Experimental design and sample size allocation (also applies to Figure 4); ^†^indicates mortality post-surgery; *n* = 5–8 mice/group. DA, dopamine; DOPAC, 3,4-dihydroxyphenylacetic acid; HPLC, high-performance liquid chromatography; IHC, immunohistochemistry; LAC, lactacystin; SN, substantia nigra; SNc, substantia nigra pars compacta; TH, tyrosine hydroxylase. Scale bar 400 μm.

Statistical analysis revealed a global effect of both LAC lesion and genotype on the number of TH + profiles in the ipsi-/contralateral SNc [lesion factor: *F*_(1,__23)_ = 16.91, *p* = 0.0004; genotype factor: *F*_(1,__23)_ = 5.62, *p* = 0.026]. *Post hoc* analyses revealed that this effect could be attributed to a significant decrease in the ratio of TH + profiles in xCT^+/+^ mice (*p* = 0.005), with no change in xCT^–/–^ mice relative to their corresponding sham (Figure 2A), resulting in a higher ratio of TH + profiles in the ipsi-/contralateral SNc of LAC-injected xCT^–/–^ mice compared to xCT^+/+^ mice (*p* = 0.036). This difference in the degree of nigral neurodegeneration translated to changes in striatal DA loss following the lesion. LAC administration led to a general decrease in DA content in the ipsi- vs. contralateral striatum that was significantly influenced by genotype [lesion × genotype factor: *F*_(1,__22)_ = 7.62, *p* = 0.011], and driven by a significant loss of DA in xCT^+/+^ mice (*p* < 0.0001), with no difference in xCT^–/–^ mice, when compared to their corresponding sham groups (Figure 2B). Similarly, LAC lesion resulted in an overall decrease of striatal DOPAC content [lesion factor: *F*_(1,__22)_ = 12.94, *p* = 0.0016], attributed to a significant decrease in xCT^+/+^ mice (*p* = 0.021), with no change in xCT^–/–^ mice compared to their corresponding sham controls (Figure 2C). No changes could be observed in the striatal DOPAC/DA ratio following the lesion [lesion factor: *F*_(1,__22)_ = 1.17, *p* > 0.05] (Figure 2D).

### Decreased Microglial Proliferation in Aged xCT-Deficient Mice Following Intranigral Administration of Lactacystin

Administration of LAC has been found to lead to widespread nigral microglial activation, observed to a higher degree in aged animals (Xiao et al., 2015; Savolainen et al., 2017). Given the role of system x_*c*_^–^ in mediating the neuroinflammatory reaction (Albertini et al., 2018), we tested its involvement in the microglial activation following LAC, and its interplay with aging, by staining SN sections of adult and aged mice for the microglial marker Iba-1.

**FIGURE 3:**
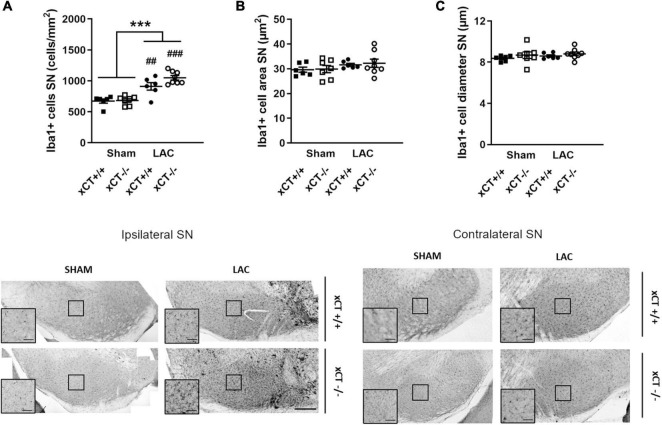
Similar neuroinflammatory reaction in the SN at 3 weeks after LAC administration in adult xCT^+/+^ and xCT^– /–^ mice. **(A)** Iba-1 immunohistochemistry revealed an increase in microglial density in the ipsilateral SN following LAC lesion, in both xCT^+/+^ and xCT^– /–^ mice. **(B,C)** No lesion-induced changes could be observed in Iba-1 + cell area **(B)** or Iba-1 + cell diameter **(C)** in xCT^+/+^ or xCT^– /–^ mice. Data are presented as mean + s.e.m. ***p < 0.001 (two-way ANOVA, lesion effect), ^##^*p* < 0.01, ^###^*p* < 0.001 (Tukey *post hoc* vs. corresponding sham group). *n* = 6–8 mice/group. LAC, lactacystin; SN, substantia nigra. Scale bar 200 μm (inset scale bar 50 μm).

In adult mice, LAC administration led to an overall increase in the density of microglial cells in the ipsilateral SN that could be observed to a similar extent in both genotypes [lesion factor: *F*_(1,__23)_ = 55.66, *p* < 0.0001], when compared to their corresponding sham groups (Figure 3A). Evaluating the morphology of the microglial cells, we failed to distinguish any difference in the Iba-1 + cell area [lesion factor: *F*_(1,__23)_ = 2.57, *p* > 0.05] (Figure 3B) or diameter [lesion factor: *F*_(1,__23)_ = 0.72, *p* > 0.05] (Figure 3C), following lesion in either genotype.

**FIGURE 4:**
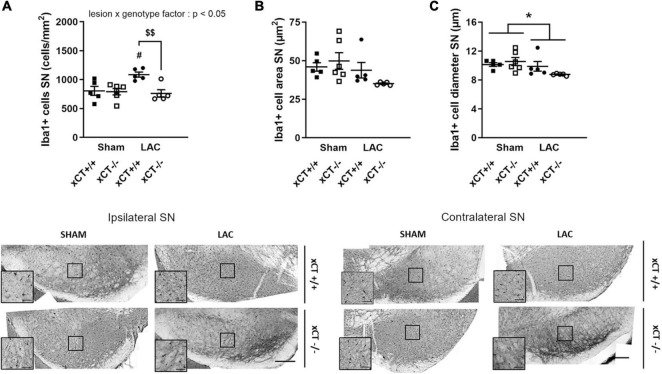
Decreased microglial reaction in the SN at 3 weeks after LAC administration in aged xCT^– /–^ mice. **(A)** Iba-1 immunohistochemistry revealed an increase in microglial density in the ipsilateral SN following LAC lesion in aged xCT^+/+^, but not xCT^– /–^ mice, when compared to their corresponding sham-injected groups. **(B,C)** No changes could be observed in Iba-1 + cell area **(B)**, while a modest general decrease of Iba-1 + cell diameter was present following lesion **(C)**. Data are presented as mean + s.e.m. *p < 0.05 (two-way ANOVA, lesion effect), ^#^*p* < 0.05 (Tukey *post hoc* vs. corresponding sham group), ^$$^*p* < 0.01 (Tukey *post hoc* comparing LAC xCT^+/+^ vs. LAC xCT^– /–^). *n* = 5–6 mice/group. LAC, lactacystin; SN, substantia nigra. Scale bar 200 μm (inset scare bar 50 μm).

In aged mice, intranigral LAC resulted in an increase in Iba-1 + microglial cell density in xCT^+/+^ mice, in presence of a significant lesion x genotype interaction factor [*F*_(1,__17)_ = 6.34, *p* = 0.022]. Our analyses revealed a ∼ 35% increase in the density of microglial profiles in the ipsilateral SN of xCT^+/+^ mice (*p* = 0.027), with no change in microglial density in xCT^–/–^ mice when compared to their corresponding sham mice (Figure 4A). LAC lesion did not significantly influence the area of microglial cells [lesion factor: *F*_(1,__17)_ = 4.10, *p* > 0.05] (Figure 4B), but did cause a slight global reduction in cell diameter in both genotypes [lesion factor: *F*_(1,__17)_ = 4.89, *p* < 0.05] (Figure 4C).

### Genetic Loss of xCT Increases the Susceptibility of Nigral Dopaminergic Neurons to 1-Methyl-4-Phenyl-1,2,3,6-Tetrahydropyridine-Induced Degeneration but Does Not Influence Striatal Dopamine Denervation in Aged Mice

To evaluate whether xCT deletion in aged mice confers protection against toxins with a distinct mechanism of action, we tested the susceptibility of aged xCT^–/–^ mice in the mitochondrial inhibition-based MPTP model.

**FIGURE 5:**
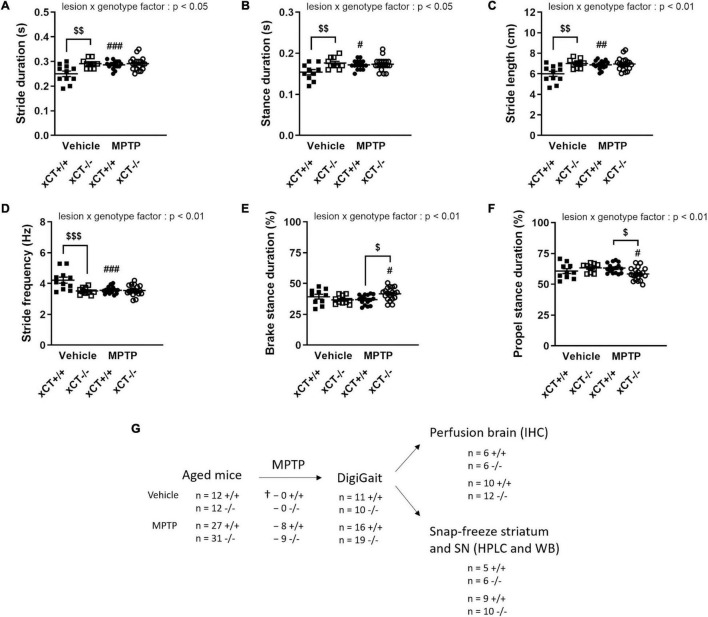
Behavioral changes following MPTP administration in aged xCT^+/+^ and xCT^– /–^ mice. **(A–D)** Administration of MPTP led to an increase in stride duration **(A)**, stance duration **(B)**, stride length **(C)**, and a decrease in stride frequency **(D)**, in aged xCT^+/+^ mice, an effect that was absent in aged xCT^– /–^ mice. **(E,F)** On the other hand, MPTP treatment led to a different composition of the stance phase in aged xCT^– /–^ mice, with an increase in the brake stance duration **(E)**, and a corresponding decrease in the propel stance duration **(F)**, an effect that could not be observed in aged xCT^+/+^ mice. Data are presented as mean + s.e.m. ^#^*p* < 0.05, ^##^*p* < 0.01, ^###^*p* < 0.001 (Tukey *post hoc* vs. corresponding vehicle group), ^$^*p* < 0.05, ^$$^*p* < 0.01, ^$$$^*p* < 0.001 (Tukey *post hoc* comparing MPTP xCT^+/+^ vs. MPTP xCT^–/–^). **(G)** Experimental design and sample size allocation (also applies to Figure 6); † indicates mortality post MPTP injection; *n* = 10–19 mice/group. HPLC, high-performance liquid chromatography; IHC, immunohistochemistry; MPTP, 1-methyl-4-phenyl-1,2,3,6-tetrahydropyridine; WB, Western blot.

Behavioral evaluation of the treated mice using the DigiGait apparatus revealed MPTP-induced changes that could be observed preferentially either in xCT^+/+^ or xCT^–/–^ mice. MPTP administration led to an overall increase in stride duration, an effect observed in presence of a significant lesion x genotype effect [*F*_(1,__52)_ = 6.86, *p* = 0.011]. In particular, MPTP was found to increase the duration of the stride by ∼15% in xCT^+/+^ mice (*p* = 0.0028), with no apparent change in xCT^–/–^ mice, when compared to their corresponding vehicle-injected mice (Figure 5A). Similarly, MPTP led to an increased stance duration, defined as the duration in which the paws are in contact with the treadmill, in xCT^+/+^, but not xCT^–/–^ mice, compared to the corresponding vehicle-treated mice [lesion × genotype factor: *F*_(1,__51)_ = 6.13, *p* = 0.017] (Figure 5B). MPTP-treated xCT^+/+^ mice also showed an increase in stride length, contrary to xCT^–/–^ mice [lesion × genotype factor: *F*_(1,__52)_ = 8.02, *p* = 0.0066] (Figure 5C), with a corresponding decrease in the stride frequency [lesion × genotype factor: *F*_(1,__52)_ = 10.81, *p* = 0.0018], that was not observed in MPTP-treated xCT^–/–^ mice (Figure 5D). For all aforementioned parameters, while MPTP had no added effect in xCT^–/–^ mice, we could measure significant differences in vehicle-injected xCT^–/–^ vs. xCT^+/+^ mice (Figures 5A–D), that mirrored changes observed in MPTP-treated xCT^+/+^ mice. This increase in stride length and duration, with the corresponding decrease in stride frequency, indicates that MPTP-treated xCT^+/+^ mice and vehicle-treated xCT^–/–^ mice demonstrated gait deficits, taking longer steps, in a longer period of time, and with a corresponding decrease in the frequency of steps, compared to vehicle-treated xCT^+/+^ mice. On the other hand, qualitative assessment of the stance revealed differences specifically in the MPTP-treated xCT^–/–^ mice. MPTP treatment led to an increase in the brake stance duration [lesion × genotype factor: *F*_(1,__51)_ = 7.56, *p* = 0.0082; ∼13% increase in xCT^–/–^, *p* = 0.048 vs. corresponding vehicle; no change in xCT^+/+^ vs. corresponding vehicle] (Figure 5E), with a corresponding decrease in the propel stance duration [lesion × genotype factor: *F*_(1,__51)_ = 7.56, *p* = 0.0082; ∼8% decrease in xCT^–/–^, *p* = 0.048 vs. corresponding vehicle; no change in xCT^+/+^ vs. corresponding vehicle] (Figure 5F), an effect that could not be observed in MPTP-treated xCT^+/+^ mice. This indicates that the MPTP-lesioned xCT^–/–^ mice spent more time in the braking portion of the stance, and less time in the propelling portion of the stance, reflecting qualitative changes in their gait during the test. As such, MPTP administration led to motor deficits in both xCT^–/–^ and xCT^+/+^ mice, with particular aspects of gait affected in each genotype.

**FIGURE 6:**
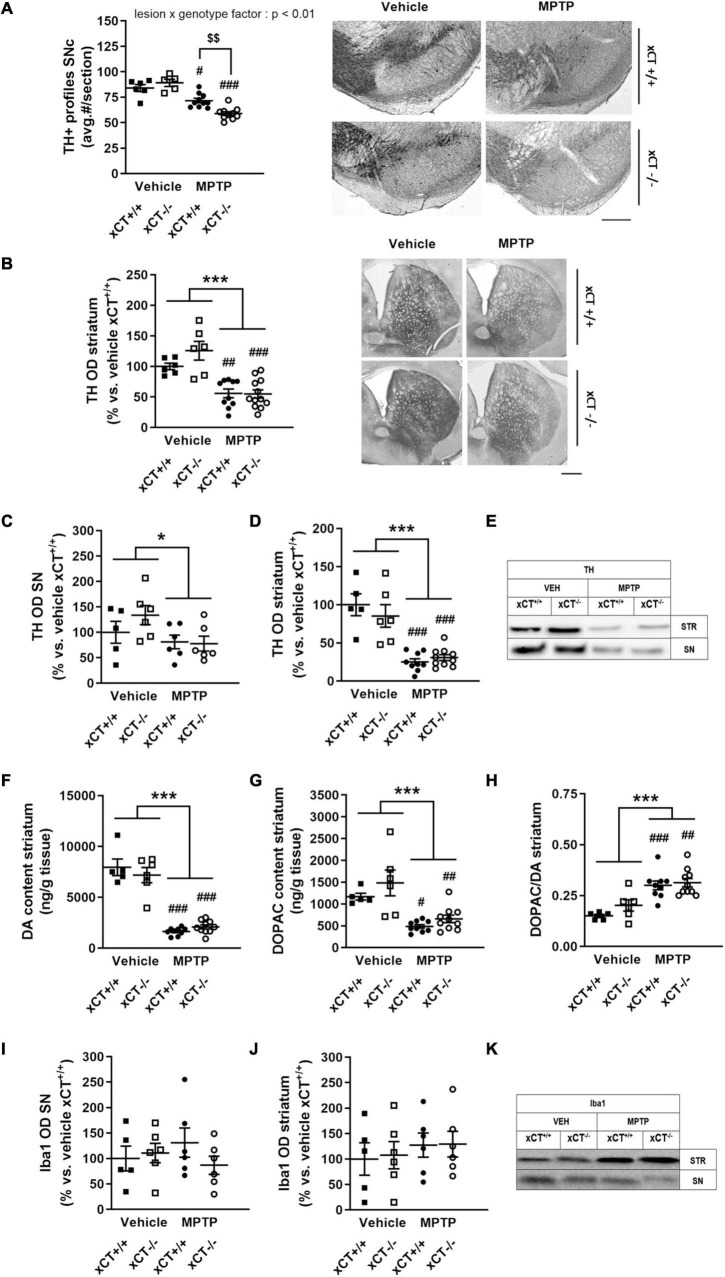
xCT deletion increases the susceptibility of nigral dopaminergic neurons for MPTP-induced degeneration in aged mice, without affecting striatal DA denervation or microglial reaction. **(A,B)** TH immunohistochemistry revealed a greater loss of nigral TH/dopaminergic neurons in xCT^– /–^ mice following chronic administration of MPTP **(A)**, but an equivalent decrease of TH + striatal innervation between genotypes **(B)**. **(C–E)** Evaluation of TH expression in SN **(C)** and striatal **(D)** extracts revealed a comparable loss of TH protein following MPTP of both genotypes. **(F)** This was reflected in a similar MPTP-induced loss of striatal DA content in xCT^– /–^ and xCT^+/+^ mice. **(G,H)** MPTP administration led to an equivalent reduction of striatal DOPAC content in both genotypes **(G)**, and an increase in striatal DOPAC/DA ratio **(H)**. **(I–K)** No significant MPTP-induced changes in expression of Iba-1 in SN **(I)** or striatum **(J)** could be revealed in either xCT^– /–^ or xCT^+/+^ mice. Data are presented as mean + s.e.m. *p < 0.05, ***p < 0.001 (two-way ANOVA, lesion effect), ^#^*p* < 0.05, ^##^*p* < 0.01, ^###^*p* < 0.001 (Tukey *post hoc* vs. corresponding vehicle group), ^$$^*p* < 0.01 (Tukey *post hoc* comparing MPTP xCT^+/+^ vs. MPTP xCT^– /–^). *n* = 5–10 mice/group in **(A)**, *n* = 6–12 mice/group in **(B)**, *n* = 5–6 mice/group in **(C)**, *n* = 5–10 mice/group in **(D–H)**, *n* = 5–6 mice/group in **(I–J)**. DA, dopamine; DOPAC, 3,4-dihydroxyphenylacetic acid; MPTP, 1-methyl-4-phenyl-1,2,3,6-tetrahydropyridine; OD, optical density; SN, substantia nigra; SNc, substantia nigra pars compacta; TH, tyrosine hydroxylase. Scale bar 400 μm.

Administration of MPTP in aged mice led to an overall reduction of nigral TH + profiles in presence of a significant lesion × genotype effect [*F*_(1,__27)_ = 11.48, *p* = 0.0022]. MPTP treatment induced a ∼15% loss of TH + profiles in xCT^+/+^ mice (*p* = 0.011), and a comparatively higher loss of ∼34% TH + profiles in xCT^–/–^ mice (*p* < 0.0001), when compared to their corresponding vehicle-injected groups (Figure 6A). In contrast, the loss of DA-ergic fibers, as evaluated by measuring the optical density of TH immunoreactivity at the level of the striatum, was observed to a similar degree in both genotypes following administration of the toxin [lesion factor: *F*_(1,__30)_ = 41.72, *p* < 0.0001] (Figure 6B). In a second group of mice, lesion degree was analyzed using Western blotting. Nigral/midbrain TH expression was shown to be reduced following MPTP in both genotypes [lesion factor: *F*_(1,__19)_ = 4.85, *p* = 0.04] (Figures 6C,E). Similarly, MPTP treatment led to a reduction of TH expression in striatal homogenates, that could be observed to a similar extent in both genotypes [lesion factor: *F*_(1,__26)_ = 56.94, *p* < 0.0001] (Figures 6D,E). This was mirrored by a similar genotype-independent loss of striatal DA content following MPTP [lesion factor: *F*_(1,__26)_ = 170.4, *p* < 0.0001], compared to the corresponding vehicle groups (Figure 6F). A loss of striatal DOPAC content could be observed to a similar extent in both genotypes [lesion factor: *F*_(1,__26)_ = 28.98, *p* < 0.0001] (Figure 6G), and MPTP administration led to an increased DOPAC/DA ratio in both xCT^+/+^ and xCT^–/–^ mice [lesion factor: *F*_(1,__26)_ = 31.33, *p* < 0.0001] (Figure 6H).

Western blotting was used to evaluate Iba-1 expression as a measure for microglial reaction to MPTP treatment, and failed to reveal any significant lesion-induced changes in either the SN/midbrain [lesion factor: *F*_(1,__19)_ = 0.02, *p* > 0.05] (Figures 6I,K), or striatal extracts [lesion factor: *F*_(1,__19)_ = 0.85, *p* > 0.05] (Figures 6J,K).

## Discussion

Despite a surge of ongoing efforts to find and validate novel neuroprotective targets in PD, the greatest pitfall remains the translation of pre-clinical findings to the clinic. The most promising therapeutic targets identified in pre-clinical research, eventually fail to fulfill their purpose in clinical trials. Multiple reasons have been proposed that might cause these problems, such as the lack of external validity of the used model or the lack of heterogeneity in pre-clinical studies, including reliance on animal models with a limited number of mechanisms of action (van der Worp et al., 2010; Athauda and Foltynie, 2015). Moreover, since PD is an age-related disease, the importance of the use of an aged animal model is highly underestimated (Reeve et al., 2014). Therefore, applying various PD mouse models to aged mice might increase the chances of successful clinical translation.

We previously reported that adult and aged mice lacking xCT were protected against 6-OHDA-induced nigral dopaminergic neurodegeneration (Massie et al., 2011), whereas adult xCT-deficient mice were equally susceptible in the systemic MPTP model (Bentea et al., 2015a). In the present study, we investigated whether loss of xCT can mitigate nigrostriatal degeneration induced by intranigral injection of the proteasome inhibitor LAC in adult and aged mice, and following systemic administration of MPTP in aged animals.

Contrary to adult mice, aged mice lacking xCT are remarkably resistant to LAC-induced nigral dopaminergic cell loss. When subjecting aged male xCT^–/–^ mice to nigral proteasome inhibition, we failed to observe any significant loss of nigral TH + neurons or striatal DA levels in the ipsilateral hemisphere. The finding that neuroprotection conferred by loss of xCT is age-dependent is intriguing and suggests that different pathways might be activated by proteasome inhibition in the aged vs. young brain. Previously, Gavilan et al. (2009) identified deficits in the activation of the unfolded protein response in the aged brain that might account for an increased sensitivity toward proteasome inhibition-induced neurodegeneration. In our study we did not observe a potentiation of the degree of lesioning following LAC with aging, however, caution must be drawn as adult and aged mice were lesioned with different batches of LAC, that may have an impact on the severity of the extent of the lesion (personal observation).

Previous findings also support the notion that administration of neuroprotective agents can lead to differential effects when evaluated in aged vs. young animals. For instance, adenovirus-mediated delivery of GDNF to the SN has decreased neuroprotective properties against intrastriatal 6-OHDA in aged vs. young rats (Choi-Lundberg et al., 1997; Connor et al., 1999). While the reason for such age-related effects is not always clear, it might be linked with a differential expression of the target (or the environment in which the target is expressed) in the aging brain. However, we failed to detect any age-related change in xCT expression in different regions of the brain, including cortex and striatum (unpublished observations). In addition, aging affects a multitude of pathways [e.g., neuroinflammation, oxidative stress, mitochondrial dysfunction, protein degradation systems (Collier et al., 2011)], which form a complex environment in which the therapeutic target should act. One of the intricate outcomes of such a complex interaction is that aged neurons become more sensitive to the toxicity of glutamate (Brewer, 1998; Brewer et al., 2005), possibly as a result of age-related depolarization of the mitochondrial membrane potential and increased production of mitochondrial reactive oxygen species (Parihar and Brewer, 2007), as well as migration of glutamate receptors to extrasynaptic sites (Avila et al., 2017). Noteworthy, system x_*c*_^–^ has been identified to play a major role in controlling extracellular glutamate levels in distinct brain regions (De Bundel et al., 2011; Massie et al., 2011). It is likely that system x_*c*_^–^—mediated glutamate release in the SN can represent a direct source of toxicity to nigral dopaminergic cell bodies causing excitotoxic cell damage in the model, especially in the context of an age-dependent decrease in the expression of glutamate reuptake transporters (Farrand et al., 2015), and redistribution of N2B-containing NMDA receptors to extrasynaptic sites with aging (Avila et al., 2017). In line with this hypothesis, intranigral administration of NMDA or the NMDA agonist quinolinic acid was found to lead to excitotoxic damage of nigral dopaminergic neurons (Connop et al., 1995), while inhibition of glutamate reuptake transporters in the SNc using L-trans-pyrrolidine-2,4-dicarboxylate similarly triggers death of nigral dopaminergic neurons *via* excitotoxic pathways, in the presence of microglial activation (Assous et al., 2014). As such, xCT deletion in the aged brain may reduce excitotoxic stress on nigral dopamine neurons leading to their increased resilience.

Qualitative differences might also exist in the reaction of the aged brain to environmental stimuli. For instance, microglial cells become primed with aging, and exhibit an exaggerated inflammatory response to secondary (even sub-threshold) challenges (Norden et al., 2015). Furthermore, chronic persistent neuroinflammation, as occurring during aging (Lynch, 2010), can synergize with proteasome inhibition and exacerbate the ensuing pathology. For instance, intra-hippocampal administration of lipopolysaccharide (LPS) 24 h prior to intra-hippocampal LAC leads to increased accumulation of ubiquitinated proteins and neurodegeneration, compared to each toxin alone (Pintado et al., 2012). Similarly, prior systemic administration of LPS to trigger brain inflammation in adult mice increases the susceptibility of the nigrostriatal pathway for LAC-induced neurodegeneration (Deneyer et al., 2019).

Interestingly, system x_*c*_^–^ has recently emerged as a novel regulator of the microglial phenotype. Cultured xCT^–/–^ primary microglial cells exposed to LPS showed reduced production of nitric oxide and release of pro-inflammatory cytokines such as TNF-α and IL-6 (Mesci et al., 2015). Furthermore, xCT^–/–^ mice demonstrate reduced peripheral and central inflammatory reaction and sickness behavior following systemic LPS administration, indicating a primary role of system x_*c*_^–^ in regulating the inflammatory response (Albertini et al., 2018). Our findings reveal decreased microglial activation in the SN of aged xCT^–/–^ mice that paralleled the neuroprotection observed in these conditions. While in this study it is difficult to temporally connect these findings, one possibility is that loss of xCT might maintain the brain in a decreased primed state during aging, lowering the subsequent reaction to toxic stimuli. Alternatively, the reduced neuroinflammatory reaction observed following LAC in aged xCT^–/–^ mice might also be the result of reduced neurodegeneration. In light of the age-dependent neuroprotection observed in xCT^–/–^ mice, it remains of interest to further investigate the impact of xCT deficiency on aging and age-related microglial priming.

In contrast with the age-dependent neuroprotection observed in the LAC model, aged xCT^–/–^ mice demonstrated an enhanced susceptibility for nigral dopaminergic neuron loss following progressive MPTP administration. Despite this increased susceptibility at the level of the cell bodies, markers of striatal dopaminergic innervation, including TH expression and DA content, decreased to a similar extent in xCT^–/–^ and xCT^+/+^ mice following MPTP treatment, potentially indicating the capacity of the remaining dopaminergic neurons to compensate for the loss of striatal innervation. Our previous findings revealed equivalent susceptibility of adult xCT^–/–^ mice to nigral dopaminergic neurodegeneration and striatal denervation following progressive administration of MPTP (Bentea et al., 2015a). Together with the present results, this indicates that nigral dopaminergic neurons of aged, but not adult, xCT^–/–^ mice show an increase in the susceptibility to the toxic effects of MPTP, which contrasts with our current observations in the LAC model and the 6-OHDA model (Massie et al., 2011). It is interesting to speculate on these dissimilar findings, and they may be related with age-related changes in mitochondrial metabolism and generation of reactive oxygen species, the distinct mechanism of action of MPTP in aged animals (Ali et al., 1994; Kuhn et al., 2003), and the nature of the induced cell loss (chronic and progressive for MPTP vs. acute for LAC and 6-OHDA). The different mechanism of action (proteasomal vs. mitochondrial inhibition) might also play a factor in the dissimilar findings in the two models, in line with the observation that targets, such as the ghrelin receptor, may differentially influence LAC- or MPTP-induced death of nigral dopaminergic neurons (Andrews et al., 2009; Coppens et al., 2017). Finally, the site of delivery (intracerebral vs. systemic) may also trigger different neuroinflammatory reactions in primed mice, that may subsequently contribute to the mechanisms of neurodegeneration. In addition, it is noteworthy that MPTP requires metabolism by monoamine oxidase B to form the active metabolite 1-methyl-4-phenylpyridinium (MPP +) in astrocytes, and further studies would be required to evaluate possible age-related changes in this enzyme in xCT^–/–^ mice.

In conclusion, we demonstrate age-dependent neuroprotection of nigral dopaminergic neurons in xCT^–/–^ mice injected with the proteasome inhibitor LAC. These results indicate that system x_*c*_^–^ is an important mediator of proteasome inhibition-induced dopaminergic neurodegeneration in aged animals and underline the need for further studies to investigate its interplay with aging, proteasomal dysfunction and mechanisms of neurotoxicity. Given the contrasting findings obtained in the progressive MPTP model, our study highlights the importance of using animal models with distinct mechanisms of action and applying them to both adult and aged animals, for profiling pre-clinical targets in PD. Future studies are required to obtain further insight into the extent to which system x_*c*_^–^ can modulate nigral cell loss in PD. In addition, exploring the therapeutic efficiency in female animals, as well as confirming the effects of the genetic knock-out using system x_*c*_^–^ inhibitors as described recently (Dang et al., 2017), will further shed light on the translatability of this approach in PD patients.

## Data Availability Statement

The raw data supporting the conclusions of this article will be made available by the authors, without undue reservation.

## Ethics Statement

The animal study was reviewed and approved by the Ethical Committee for Animal Experimentation Vrije Universiteit Brussel, and Portland VA Medical Center Institutional Animal Care and Use Committee.

## Author Contributions

EB, HS, AV, CM, and AM designed the experiments. EB, LDP, LV, LW, LD, CM, and GA performed the experiments. EB, LDP, LV, AV, CM, and AM analyzed and interpreted data. EB, LDP, CM, and AM wrote the manuscript. All authors discussed the results, edited and commented on the article.

## Conflict of Interest

The authors declare that the research was conducted in the absence of any commercial or financial relationships that could be construed as a potential conflict of interest.

## Publisher’s Note

All claims expressed in this article are solely those of the authors and do not necessarily represent those of their affiliated organizations, or those of the publisher, the editors and the reviewers. Any product that may be evaluated in this article, or claim that may be made by its manufacturer, is not guaranteed or endorsed by the publisher.
